# First-in-human pilot study of an integrin α6-targeted radiotracer for SPECT imaging of breast cancer

**DOI:** 10.1038/s41392-020-00266-9

**Published:** 2020-08-11

**Authors:** Shi Gao, Bing Jia, Guokai Feng, Chengyan Dong, Hui Du, Lin Bai, Qian Zhong, Qingjie Ma, Musheng Zeng, Fan Wang

**Affiliations:** 1 Department of Nuclear Medicine, China–Japan Union Hospital, Jilin University, 130033 Jilin, China; 2 Medical Isotopes Research Center and Department of Radiation Medicine, State Key Laboratory of Natural and Biomimetic Drugs, School of Basic Medical Sciences, Peking University, 100191 Beijing, China; 3 State Key Laboratory of Oncology in South China, Collaborative Innovation Center for Cancer Medicine, Sun Yat-sen University Cancer Center, 510060 Guangzhou, China; 4 Key Laboratory of Protein and Peptide Pharmaceuticals, CAS Center for Excellence in Biomacromolecules, Institute of Biophysics, Chinese Academy of Sciences, 100101 Beijing, China; 5 GE Healthcare China, 100176 Beijing, China; 6 Bioland Laboratory (Guangzhou Regenerative Medicine and Health Guangdong Laboratory), 510005 Guangzhou, China

**Keywords:** Cancer imaging, Tumour biomarkers

**Dear Editor,**

The molecular classification has been playing a crucial role in the precise theranostics of cancer. Compared with the invasive biopsy, the in vivo noninvasive detection of biomarkers by nuclear imaging possesses advantages due to tumor heterogeneity. As one member of the integrin family, integrin α6 subunit combines the integrin β1 or β4 subunit to form integrin α6β1 or α6β4 heterodimers, which function to promote the migration, invasion, and survival of tumor cells, leading to increased metastasis, poor prognosis, and reduced survival. Therefore, the in vivo imaging of integrin α6 expression could play an important role in the treatment planning and prognosis prediction.

Integrin α6 is overexpressed on breast cancer cells^1^ as well as activated endothelial cells during breast cancer angiogenesis.^2^ High expression of integrin α6 significantly correlated with increased mortality risk and reduced overall survival in a clinical study of 119 patients with invasive breast cancer.^3^ In our previous study, we identified an integrin α6-targeted peptide CRWYDENAC (named RWY)^4^ and synthesized a positron emission tomography (PET) radiotracer ^18^F-RWY for the detection of hepatocellular carcinoma (HCC)^5^ in animal models. Considering the more cost-effective and broadly available single photon emission computed tomography/computed tomography (SPECT/CT) technology in clinical applications, in this study, we labeled the RWY peptide with radionuclide ^99m^Tc to prepare a radiotracer ^99m^Tc-RWY for SPECT imaging of breast cancer.

^99m^Tc-RWY was prepared using the tricine/EDDA system (Supplementary Fig. 1a). After radiolabeling, the radiochemical purity of ^99m^Tc-RWY calculated by radio-HPLC was >98% (Supplementary Fig. 1b). The radiolabeling procedure was simple, efficient, and reproducible, enabling the development of a kit formulation and easy availability for routine clinical use. The radiotracer was stable in saline for more than 6 h, and >90% of the original form was retained in the urine sample collected at 1 h post-injection (p.i.) (Supplementary Fig. 1c, d). In general, ^99m^Tc-RWY remained stable and exhibited rapid clearance from the major organs, predominantly via the renal route (Supplementary Fig. 1e and f). With the exception of kidney uptake (4.48 ± 1.29 %ID/g), the accumulation of ^99m^Tc-RWY in the major organs did not exceed 0.40 %ID/g at 1 h p.i. (Supplementary Fig. 1g). The rapid clearance rate and low background in normal organs would be a benefit for the in vivo imaging.

^99m^Tc-RWY/SPECT was performed in different animal models preclinically. Both integrin α6-positive MDA-MB-231 and SK-BR3 tumors were clearly visualized, while integrin α6-negative S18sh tumors had no tracer accumulation (Supplementary Fig. 1h). These findings suggested that ^99m^Tc-RWY had excellent in vivo specific targeting properties.

We further assessed the toxicity of unlabeled RWY peptide and ^99m^Tc-RWY in mice, and the results are shown in Supplementary Figs. 2–4. Given the excellent targeting properties and safety of ^99m^Tc-RWY in mice, we further translated this SPECT radiotracer into clinical studies. The first-in-human pilot study of ^99m^Tc-RWY/SPECT imaging was conducted in seven healthy volunteers, including four males and three females (Supplementary Table 1). Series of planar whole-body SPECT images of a representative subject showed the distribution of ^99m^Tc-RWY between 30 min and 24 h p.i. (Supplementary Fig. 5). Except for the intense accumulation in the kidneys and bladder corresponding to the main excretion pathway, there was moderate ^99m^Tc-RWY uptake in the heart and nasal cavity at 30 min p.i. Compared with the images obtained at 30 min p.i, the images at 1 h p.i. showed much lower background in the chest, which may benefit the imaging for breast tumors. Low liver uptake and background signals may allow for easy screening of metastases in the liver and other organs. The quantitative data are shown in Supplementary Fig. 6.

The summary of dosimetry parameters of ^99m^Tc-RWY for various organs and the whole body are analyzed (Supplementary Table 2). The mean effective dose equivalent was (2.99 ± 0.39) × 10^−3^ mSv/MBq, and the effective radiation dose to the body was 2.34 ± 0.05 mSv. Compared with other ^99m^Tc-labeled clinical agents, such as methylene diphosphonate (MDP) for bone check (3.4 mSv), tetrofosmin sestamibi for myocardial perfusion (4.2–4.6 mSv) and hexamethylpropylene-amineoxime (HMPAO) for the brain (7.4 mSv), the effective radiation dose of ^99m^Tc-RWY (2.34 ± 0.05 mSv) was significantly lower.

After evaluating the safety of ^99m^Tc-RWY in animals and healthy volunteers, we conducted the pilot clinical study in two breast cancer patients to investigate the potential clinical application of integrin α6-targeted imaging. In patient #1, who was confirmed later as the clinical stage II breast cancer in the left breast, a SPECT/CT scan was acquired 30 min after the intravenous administration of ^99m^Tc-RWY. In the transverse plane CT image (Fig. 1a), a significant area of increased density was identified in the left breast compared with the area in the right breast. The transverse plane SPECT/CT image (Fig. 1b) displayed intense focal radiotracer uptake in the suspected tumor area. Without the interference of signals from other organs, the coronal plane SPECT/CT images (Fig. 1c, d) and the sagittal plane SPECT/CT image (Fig. 1e, f) displayed a more favorable tumor imaging effect with a low background. Representative hematoxylin–eosin (HE) staining of the high uptake region from the left breast showed the presence of tumor cells (Fig. 1g), and the immunohistochemical staining showed positive integrin α6 expression (Fig. 1h).

**Fig. 1:**
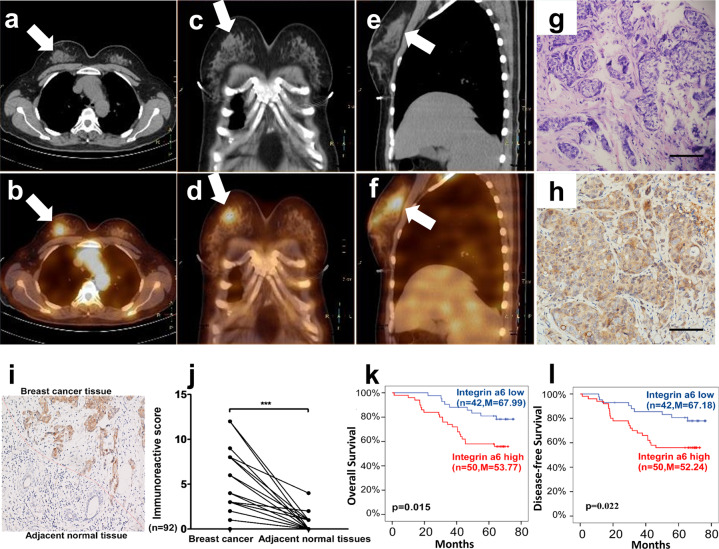
^99m^Tc-RWY SPECT/CT imaging of a 52-year-old woman (patient 1#) with clinical stage II breast cancer in the left breast and the expression level and the clinical significance of integrin α6 in breast cancer. **a**, **b** Transverse, **c**, **d** coronal, and **e**, **f** sagittal plane CT and SPECT/CT. SPECT/CT images of the chest obtained 0.5 h p.i. displayed intense ^99m^Tc-RWY accumulation in the cancer tissue in the left breast (white arrow). **g** Hematoxylin–eosin (HE) staining confirmed the presence of tumor cells in the section indicated by the white arrow. Scale bar, 100 µm. **h** Immunohistochemical staining indicated high integrin α6 expression in the area with high radioactivity accumulation. We also tested the expression and prognostic significance of integrin α6 in breast cancer patients. Scale bar, 100 µm. **i** A representative image showed varied integrin α6 expression between the breast cancer tissue and the adjacent normal tissue. **j** The integrin α6 was expressed at a higher level in breast cancer tissues than that in adjacent normal tissues. Ninety-two breast cancer tissues and their adjacent normal tissues were analyzed. The statistical analysis was performed using the paired *t* test, ***p < 0.001. **k**, **l** High integrin α6 expression (Immunoreactive score (IRS) > 4.0) correlated with poor prognosis. Patients were divided into two groups (low and high) based on their levels of integrin α6 expression with IRS = 4.0 as the cutoff value. Note that even in the low group, the breast cancer tissues still expressed a higher level of integrin α6 than the corresponding adjacent normal tissues. Kaplan–Meier plots showed significant differences in survival between the two groups. High integrin α6 expression correlated with poor prognosis for overall survival **k** and disease-free survival **l** in these patients. *n* number of patients, *M* median months of survival

In patient #2, a neoplasm in the left breast was suspected to be a tumor on the transverse plane CT image (Supplementary Fig. 7a). Following an intravenous injection of ^99m^Tc-RWY, SPECT/CT images were acquired at 60 min p.i. The intense signals observed in the SPECT/CT images (Supplementary Fig. 7b–f) showed a significant overlap for the neoplasm. A biopsy was harvested by aspiration, and breast cancer was confirmed by HE staining (Supplementary Fig. 7g). The tumor was further characterized as clinical stage III. Immunohistochemical staining of the sample from the high radiotracer accumulation region showed high integrin α6 expression (Supplementary Fig. 7h). The biochemical analyses of blood samples from the two patients before and after administration of ^99m^Tc-RWY also confirmed the safety of this new tracer (Supplementary Table 3).

To verify the expression level and the clinical significance of integrin α6 in breast cancer, we examined integrin α6 expression in 92 breast cancer tissues and the adjacent normal tissues by immunohistochemistry (Supplementary Table 4). We observed higher levels of integrin α6 expression in almost all breast cancer tissues than that in the paired adjacent normal tissues (Fig. 1i, j). The patients were divided into two groups (integrin α6 low and high) by setting the median integrin α6 expression level observed in cancer tissues as the cutoff value (IRS = 4). It was found that even in the low group, the breast cancer tissues still expressed a higher level of integrin α6 than the corresponding adjacent normal tissues. Patients with high integrin α6 expression had poorer prognosis for overall survival (Fig. 1k) and disease-free survival (Fig. 1l). These findings suggest that integrin α6 is an attractive molecular imaging target for the staging and prognosis of breast cancer.

We developed a novel integrin α6-targeted SPECT radiotracer ^99m^Tc-RWY for breast cancer imaging. The preclinical and primary clinical studies demonstrated the safety and feasibility of ^99m^Tc-RWY for further clinical translation, and the successful imaging in breast cancer patients indicated the potential values of ^99m^Tc-RWY on the diagnosis, staging, prognosis as well as guiding targeted therapy for human breast cancer and other integrin α6-positive cancers.

Supplementary information accompanies the manuscript on the *Signal Transduction and Targeted Therapy* website http://www.nature.com/sigtrans. The authors declare that all data supporting the findings of this study are available within the paper and its supplementary information files.

## Supplementary information

Supplementary Materials
