# Supplementary material for: First-in-human pilot study of an integrin α6-targeted radiotracer for SPECT imaging of breast cancer
Source: Signal Transduct Target Ther. 2020 Aug 11;5:147. doi: 10.1038/s41392-020-00266-9 (PMC7419287; doi:10.1038/s41392-020-00266-9)
Supplement: Supplementary file 1 — Supplementary Materials [file 41392_2020_266_MOESM1_ESM.docx]

Supplementary Materials for

**First-in-human pilot study of an integrin α6-targeted radiotracer for SPECT imaging of breast cancer**

**Shi Gao^1, #^, Bing Jia^2, #^, Guokai Feng^3, #^, Chengyan Dong^4, 5,#^, Hui Du^3^, Lin Bai^1^, Qian Zhong^3^, Qingjie Ma^1,^ *, Musheng Zeng^3,^ *, Fan Wang^2, 4, 6^***

^1^Department of Nuclear Medicine, China-Japan Union Hospital, Jilin University, Jilin 130033, China.

^2^Medical Isotopes Research Center and Department of Radiation Medicine, State Key Laboratory of Natural and Biomimetic Drugs, School of Basic Medical Sciences, Peking University, Beijing 100191, China.

^3^State Key Laboratory of Oncology in South China, Collaborative Innovation Center for Cancer Medicine, Sun Yat-sen University Cancer Center, Guangzhou 510060, China.

^4^Key Laboratory of Protein and Peptide Pharmaceuticals, CAS Center for Excellence in Biomacromolecules, Institute of Biophysics, Chinese Academy of Sciences, Beijing 100101, China.

^5^GE Healthcare China, Beijing 100176, China.

^6^Bioland Laboratory (Guangzhou Regenerative Medicine and Health Guangdong Laboratory), Guangzhou 510005, China.

Correspondence to: wangfan@bjmu.edu.cn

**This PDF file includes:**

Materials and Methods

Supplementary Text

Figures. S1 to S7

Tables S1 to S4

Materials and Methods

**Synthesis of HYNIC-RWY**

A solution of RWY (10 mg, 8.64 μmol) was mixed with HYNIC-NHS at a mole ratio of 1:1.2. The pH was adjusted to 8.5-9.0 using DIPEA. The reaction mixture was subsequently stirred for approximately 12 h at 40ºC. The product was isolated from the reaction mixture by HPLC purification. The products were collected and lyophilized, and the yield was approximately 41.5%.

**Preparation and preclinical evaluation of ^99m^Tc-RWY**

The following solutions were added to a clean vial: 10 μL of HYNIC-RWY solution (1 mg/mL in H_2_O), 100 μL of tricine solution (100 mg/mL in 0.1 M succinic buffer, pH 5.0), 25 μL of SnCl_2_ solution (1.0 mg/mL in 0.1 N HCl), and 100 μL of Na^99m^TcO_4_ solution (~370 MBq). The reaction mixture was maintained at 100ºC for 10 min. With the addition of 250 μL of EDDA (60 mg/mL, pH 7.0), the vial containing the reaction mixture was sealed, cramped, and heated at 100°C for 25 min. After cooling to room temperature, a sample of the resulting solution was analyzed by radio-HPLC. Then the in vitro and in vivo stability studies were performed and analyzed with radio-HPLC (details in supplementary materials).

The whole-body radioactivity clearance, blood clearance, biodistribution and toxicity of ^99m^Tc-RWYwere determined with BALB/c mice. More procedure details were introduced in supplementary materials. The preclinical SPECT/CT imaging was performed using female BALB/c nude mice bearing integrinα6-positive MDA-MB-231 and SK-BR3 tumors, as well as integrinα6-negative S18sh tumor. Each tumor-bearing mouse was anesthetized with isoflurane and administered intravenously with 18.5 MBq (37MBq/1 μg) of ^99m^Tc-RWY in 100 μL of saline. The tumor-bearing mice were imaged using NanoSPECT/CT (Mediso Ltd. Hungary). All animal experiments were performed in accordance with the guidelines from Institutional Animal Care and Use Committee at Peking University.

***In vitro* stability**

The in vitro stability of ^99m^Tc-RWY was determined by incubating the purified radiotracer in saline at room temperature. The radiochemistry purity of the samples (N = 3) incubated for 1, 2, 4 and 6 h was measured by radio-HPLC.

***In vivo* stability**

The metabolic stability of ^99m^Tc-RWY was evaluated using BALB/c nude mice. Each mouse received radiotracer (~29.6MBq) dissolved in 0.2 mL saline via the tail vein. Urine samples were collected at 1 h p.i. by manual voiding and were mixed with an equal volume of saline. The mixture was centrifuged at 8000 rpm for 5 min. The supernatant was collected and filtered through a 0.22-μm Millex-LG syringe driven filter unit to remove the precipitate. The filtrate was analyzed via radio-HPLC.

**Whole-body clearance kinetics**

Each BALB/c mouse received the radiotracer (~29.6MBq) dissolved in 0.2 mL saline via the tail vein. Using clean plastic tubes as containers, the radioactivity of each mouse was measured in a γ-counter (Wallac 1470-002, Perkin Elmer, Finland) (N = 6). Data are expressed as percentages normalized to the injected dose.

**Blood clearance kinetics**

Male and female BALB/c mice received an intravenous injection of 0.37 MBq^99m^Tc-RWY under anesthesia (n = 7). Venous blood samples (~0.01 mL) were collected at 1, 3, 5, 7, 10, 15, 20, 30, 45, 60, 90, 120, 150, 180, and 240 minp.i. The collected blood samples were weighed and counted in a γ-counter. The percentage of the injected dose per gram (%ID/g) at each time point was calculated after radionuclide decay correction.

**Biodistribution**

BALB/c mice were randomly divided into four groups of four animals each. Each mouse received ^99m^Tc-RWY (~185 kBq) dissolved in 0.1 mL of saline via the tail vein. The animals were anesthetized by isoflurane inhalation and sacrificed by cervical dislocation at 0.5, 1, 2, and 4 h p.i.The organs of interest (blood, heart, liver, spleen, kidney, stomach, and intestine) were harvested, weighed, and measured for radioactivity in a γ-counter. Organ uptake was calculated as a percentage of the injected dose per gram of the tissue mass (%ID/g). The biodistribution data were reported as the average and standard deviation (mean ± SD) based on the results from four animals at each time point.

**Toxicity study on ^99m^Tc-RWY**

BALB/c mice were fed under normal conditions and randomly divided into two groups. Three male mice and 3 female mice were injected with a single high dose (1850 MBq/kg) and a single moderate dose (185 MBq/kg) of ^99m^Tc-RWY. Two male mice and 2 female mice were injected with 0.5 mL saline. The entire injection procedure should be completed in 5 seconds. The mice in the two groups were observed and weighed. At 72 h p.i., the mice were sacrificed, and the status of the major organs was determined.

**Toxicity study on RWY peptide**

A total of 2 treatment groups of 3 male and 3 female mice each were administered with the unlabeled RWY at dose levels of 16.67 μg/kg or 166.7 μg/kg, which was 100-fold or 1000-fold higher dose than the average clinical injection amount of female subjects. A vehicle control group was also set for better comparison. All injections were dissolved in phosphate-buffered saline (PBS, pH 7.4) and the final injection volume was 100 μL for all animals through an intraperitoneal route. Observations for morbidity, mortality, injury, and weight change were conducted twice daily for a week. Then the tissues were microscopically examined.

**Cell culture and animal model**

Integrin α6-positive MDA-MB-231 and SK-BR3 human breast cancer cells were obtained from the American Type Culture Collection (Manassas, VA). The integrin α6-negative S18sh cells were obtained from Prof. Zheng’s lab. All cells were cultured in high glucose Dulbecco’s Modified Eagle’s Medium (DMEM) with 10% fetal bovine serum (FBS) at 37 °C in a humidified atmosphere containing 5% CO_2_. Female BALB/c nude mice (4-5 weeks of age) were purchased from the Department of Experimental Animals, Peking University Health Science Center. Tumor cells (2 × 10^6^) were implanted subcutaneously into the right upper flanks of the nude mice. When the tumors reached a mean diameter of ~0.8 cm, the tumor-bearing mice were used for the SPECT imaging studies.

**Subject Recruitment**

The clinical investigation of the novel radiotracer, ^99m^Tc-RWY, was approved by the ethics committees and the Institutional Review Boards (IRB) of China-Japan Union Hospital of Jilin University (Changchun, China). 7 healthy volunteers were recruited from China-Japan Union Hospital in Changchun, consisting of four male and three female Asians aged between 23 and 36 years old. Physical examination and laboratory results from the last 6 months demonstrated no pathologic findings for all volunteers. In addition, 2 female patients with suspicious breast cancer were also considered eligible for the study. Informed written consents were obtained from all 9 subjects before the procedure (ClinicalTrials.gov ID: NCT04289532). The study protocol for healthy volunteers and patients was carried out according to our previous report[^37^](#_ENREF_37).

**^99m^Tc-RWY Evaluation in Healthy Volunteers**

Seven healthy volunteers were administered with ^99m^Tc-RWY via a single intravenous bolus injection (11.1 MBq/kg), followed by a 10-mL saline flush. The planar scans were performed at 0.5 h, 1 h, 2h, 4h and 24h post-injection (p.i.). All subjects were in a supine position during imaging using a double-head γ camera (Precedence; Philips Healthcare) equipped with low-energy, parallel hole collimators. The matrix was 128 × 128 pixels, and the photopeak was centered at 140 keV with asymmetrical 20% window. Imaging was carried out using six angular steps with a 20-s time frame. The distance between the breast and the detector was minimized.

The blood sampling and urine sampling were also obtained according to our previous report[^37^](#_ENREF_37). Vital signs (body temperature, systolic and diastolic blood pressure and pulse rate), laboratory safety tests (renal and liver function chemistry, hematology, and blood coagulation parameters) and 12-lead electrocardiogram were measured before and after ^99m^Tc-RWY injection. The 1.5 mL of blood sampling was harvested at 1, 3, 5, 10, 15, 30, 60 and 120 min p.i. via an indwelling catheter, weighed, and measured for radioactivity in a γ-counter (Wallac 1470-002, Perkin-Elmer, Finland). Decay corrected time-activity curve was calculated as a percentage of the injected dose per gram of the tissue mass (%ID/g). The urine samples were collected at the following hourly intervals after tracer injection: 0 to 2, 2 to 4, 4 to 8, 8 to 12 and 12 to 24 hours, weighed for volume and measured for radioactivity in a γ-counter.

Visual analysis was applied to determine the integral biodistribution of the tracer. For each subject, regions of interest (ROIs) were delineated over the identified organs, including lung, heart, liver, kidneys, spleen, intestine, urinary bladder and a background region near the body on the anterior image. The mirror ROIs were applied to the posterior images of each organ. The mean count of each organ on planar images was measured and the results were expressed as the percentage of initial injected activity after decay-correction. Fitted residence time functions were integrated analytically to determine the area under the curve (AUC) to yield the residence time of each organ. Then, these residence times were input in OLINDA/EXM 1.0 software (Vanderbilt, University, Nashville, TN) to calculate equivalent organ doses and the effective dose (ED) based on the 70kg reference adult phantom in International Commission on Radiological Protection (ICRP) publication.

**SPECT/CT Imaging in Patients with Suspicious Breast Tumors**

The 2 female patients (Patient 1: 52 yo; Patient 2: 51 yo) with suspicious breast cancer were enrolled in this study. ^99m^Tc-RWY was also injected via a single intravenous bolus injection (11.1 MBq) followed by a 10-mL saline flush, then SPECT/CT scans of the chest were performed at 30-60 min p.i. with the similar acquired conditions as above.

Biopsies were obtained at least 7 days prior to surgery. The sections were processed in citrate buffer (pH=6) and microwaved for antigenic retrieval. Following treatment with 3% hydrogen peroxide in methanol to quench the endogenous peroxidase activity, the sections were incubated with 1% bovine serum albumin to block nonspecific binding. The sections were subsequently stained with anti-integrin α6 antibody (Abcam, ab20142, 1:50) for 12 hours at 4ºC. After washing, the sections were incubated with an HRP-conjugated polyclonal secondary antibody (1/200). The sections were immersed in 3-amino-9-ethyl carbazole and counterstained with 10% Mayer’s hematoxylin, dehydrated, and mounted in crystal mount. Integrin α6 expression in breast cancer tissues and adjacent normal tissues was characterized by two annotations, intensity and quantity, and both of them were transformed from ordinal form to numeric form. Four values strong, moderate, weak, and negative that are used to describe intensity were transformed into 3, 2, 1, and 0, respectively. Similarly, five values˃75%, 75%–51%, 50%–26%, 25%–1%, and negative that are used to describe quantity were transformed into 4, 3, 2, 1, and 0, respectively. Immunoreactive score (IRS) is calculated using intensity× quantity.

**Integrin α6 expression in breast cancer patients**

The 92 invasive breast cancer tissue samples, containing tumor tissues and adjacent normal tissues, were collected from the Department of Pathology, Sun Yat-sen University Cancer Center (Guangzhou, China). The Institutional Review Board (IRB) approval was obtained from SunYat-Sen University Cancer Center (Guangzhou, China), and the informed written consents were signed by patients prior to the use of these clinical materials for research purposes. Collected samples were fixed with formalin, embedded in paraffin, sectioned, and stained with H&E according to standard histopathological techniques. Paraffin sections were de-paraffinized with xylene and rehydrated. The detailed IHC procedure was same as above. IRS scores were statistically analyzed with Student’s paired t tests to compare the expression level of integrin α6 between breast cancer tissues and adjacent normal tissues. Furthermore, the survival information of these patients was pursued and the median IRS (IRS = 4) was set as the cutoff value for the survival analysis.

**Statistical analysis**

Data are expressed as mean ± SD, and P values of <0.05 were considered statistically significant. The statistical analysis was performed using Student’s t test.

.

Supplementary Text

Financial Support

This research was supported by grants from the National Natural Science Foundation of China (NSFC) (projects 81630045, 81927802, 81202137, 91440106, 81771869, 81871384 and 81602364), the National Key R&D Program of China (2017YFA0505600, 2016YFA0502100 and 2017YFA0205600), Strategic Priority Research Program of the Chinese Academy of Sciences (XDA12020110), the Science & Technology Project of Guangdong Province (2017A020211010), and the Health & Medical Collaborative Innovation Project of Guangzhou City, China (201400000001 and 20150802024).


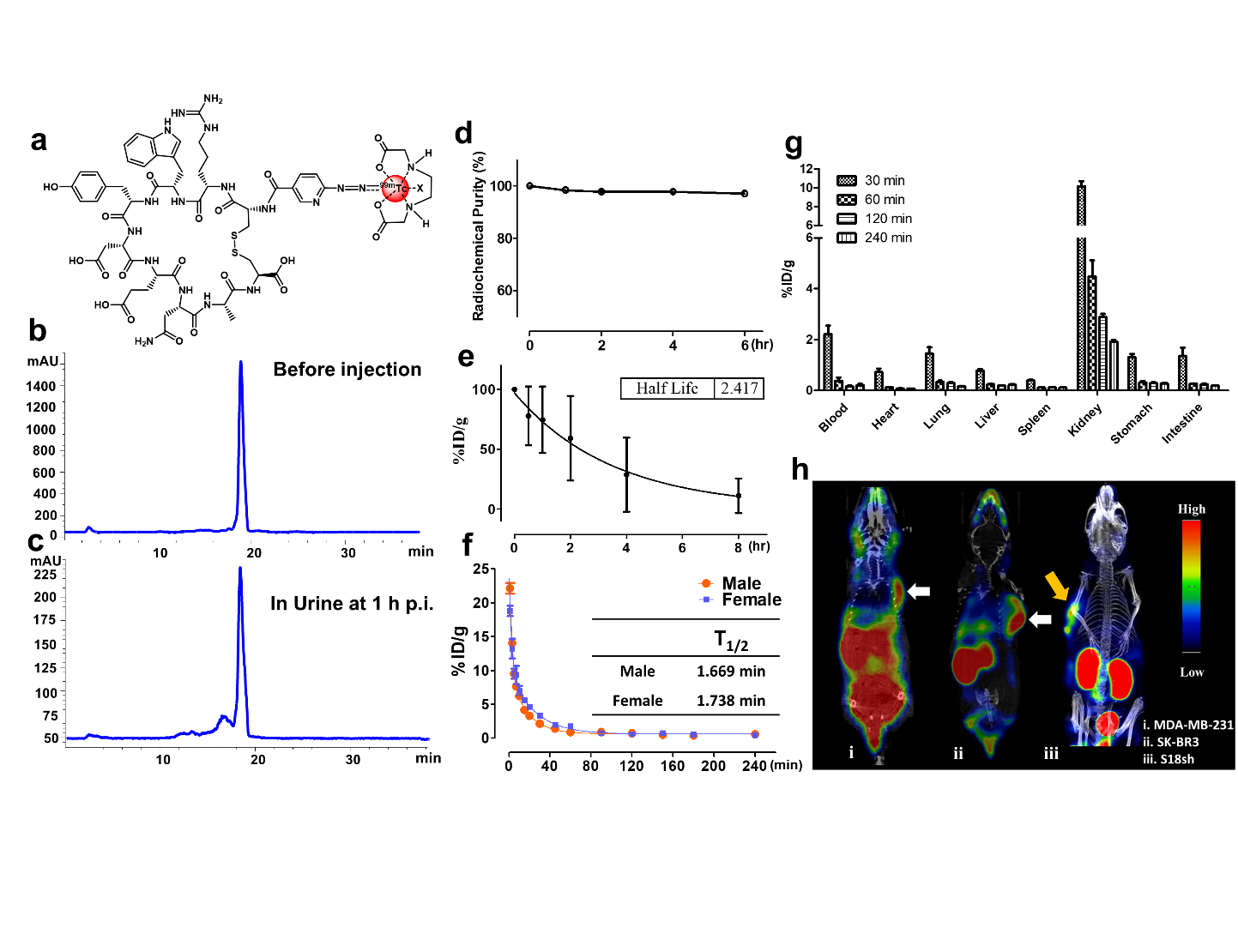


Figure. S1.

**The development of an integrin α6-targeted radiotracer ^99m^Tc-RWY.** (**a**) Structure of ^99m^Tc-RWY. (**b**) The labeling yield of ^99m^Tc-RWY is more than 98%. (**c**) In vivo stability study showed that more than 90% of the ^99m^Tc-RWY was intact in urine at 1 h p.i. (**d**) In vitro stability of ^99m^Tc-RWY in saline at room temperature for 6 hours. (**e**) The whole-body radioactivity curve of mice injected with ^99m^Tc-RWY with a general half-life of 2.417 h (n = 6). (**f**) The blood clearance curve of^99m^Tc-RWY in male and female mice over 4 h (n = 7). (**g**) Biodistribution of ^99m^Tc-RWY in mice with an intravenous injection of ^99m^Tc-RWY (~185 kBq).Organ uptake was calculated as a percentage of the injected dose per gram of wet tissue mass (%ID/g). (**h**) SPECT/CT imaging of nude mice bearing the different cancer xenografts at 1 h after the injection of ^99m^Tc-RWY (~18.5 MBq). i, integrin α6-positive MDA-MB-231breast cancer; ii, integrin α6-positive SK-BR3breast cancer; iii, integrin α6-negativeS18sh tumor.

**
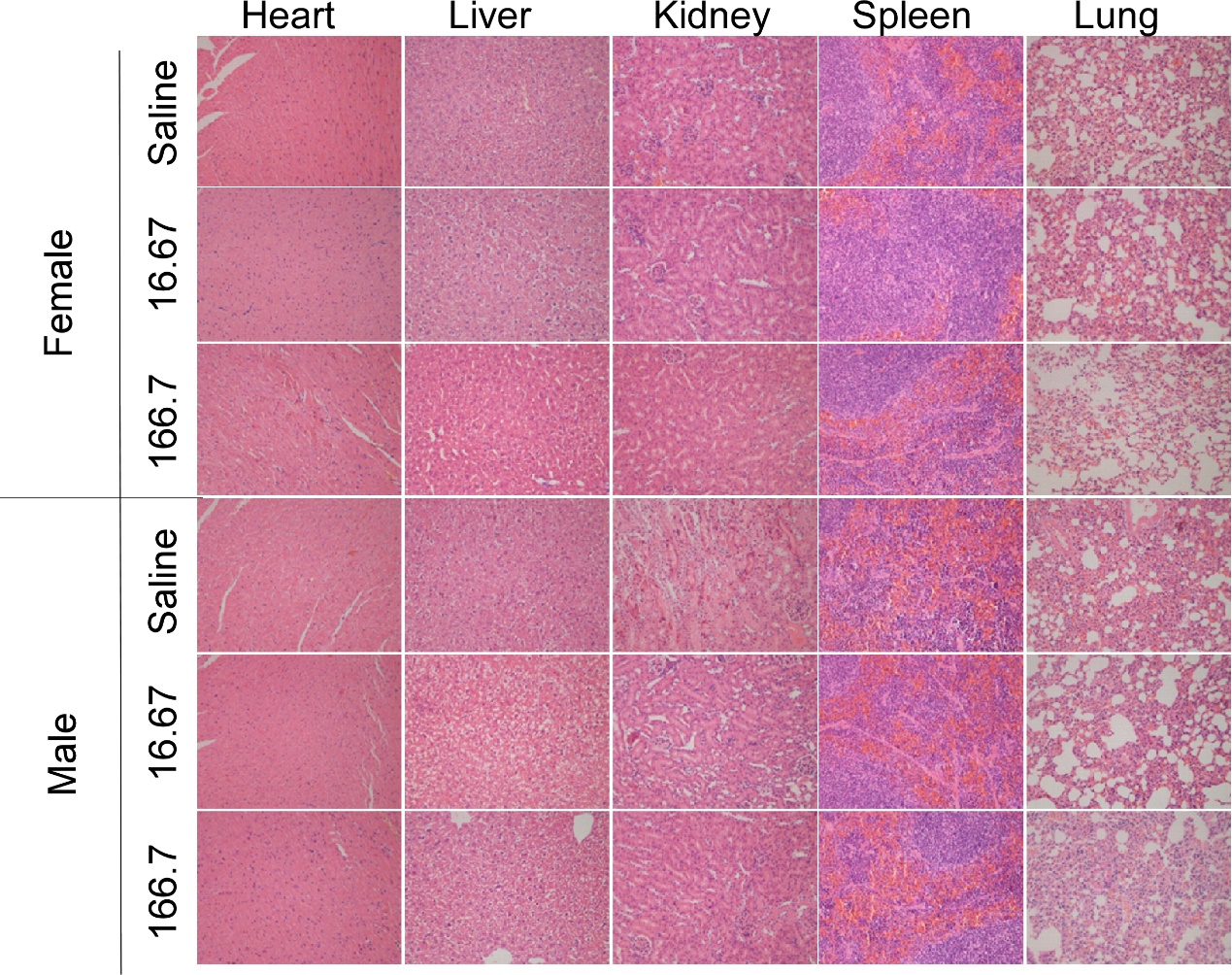
**

Figure. S2.

**HE staining of major tissues from mice injected with saline and different doses of RWY.** BALB/c mice were administered with the unradiolabeled RWY peptide at the 100-fold (16.67 μg/kg) or 1000-fold(166.7 μg/kg) higher dose than that injected in a female patient according to a previous report([*2*](#_ENREF_2)) Compared with the saline group, no histological changes were observed in the major organs of mice injected with the excess peptide dose


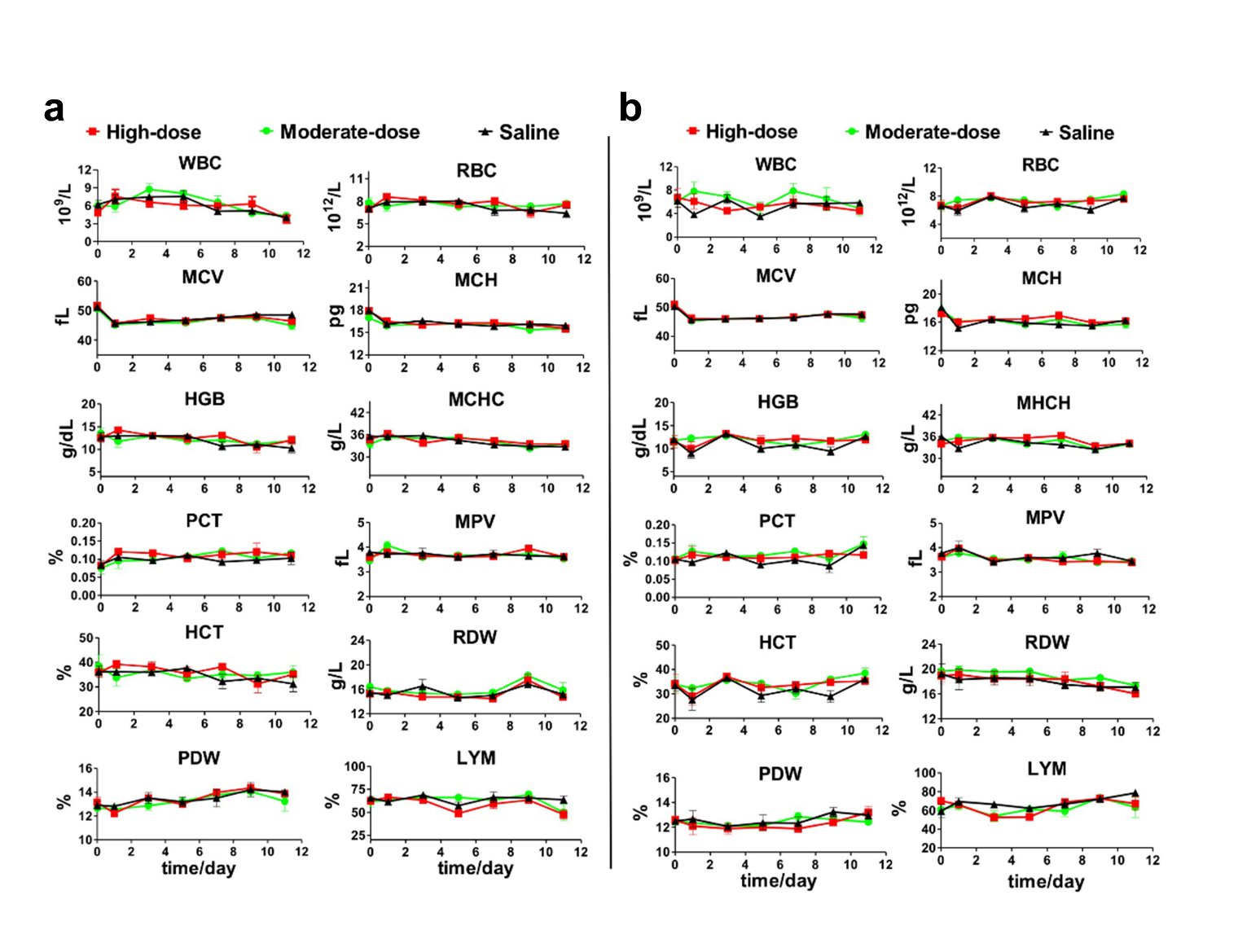


Figure. S3.

**Analyses of blood samples from mice injected with saline and different doses of ^99m^Tc-RWY.** (**a**)Female mice and (**b**) male mice in the moderate dose group were injected with 185 MBq/kg ^99m^Tc-RWY, and mice in the high dose group were injected with 1850 MBq/kg ^99m^Tc-RWY (normal dose: 74 MBq/kg calculated for this species). WBC: white blood cell; RBC: red blood cell; MCV: mean corpuscular volume; MCH: mean corpuscular hemoglobin; HGB: hemoglobin; MCHC: mean corpuscular hemoglobin concentration; PCT: procalcitonin; MPV: mean platelet volume; HCT: hematocrit; RDW: red cell distribution width; PDW: platelet distribution width; LYM: lymphocyte.

**
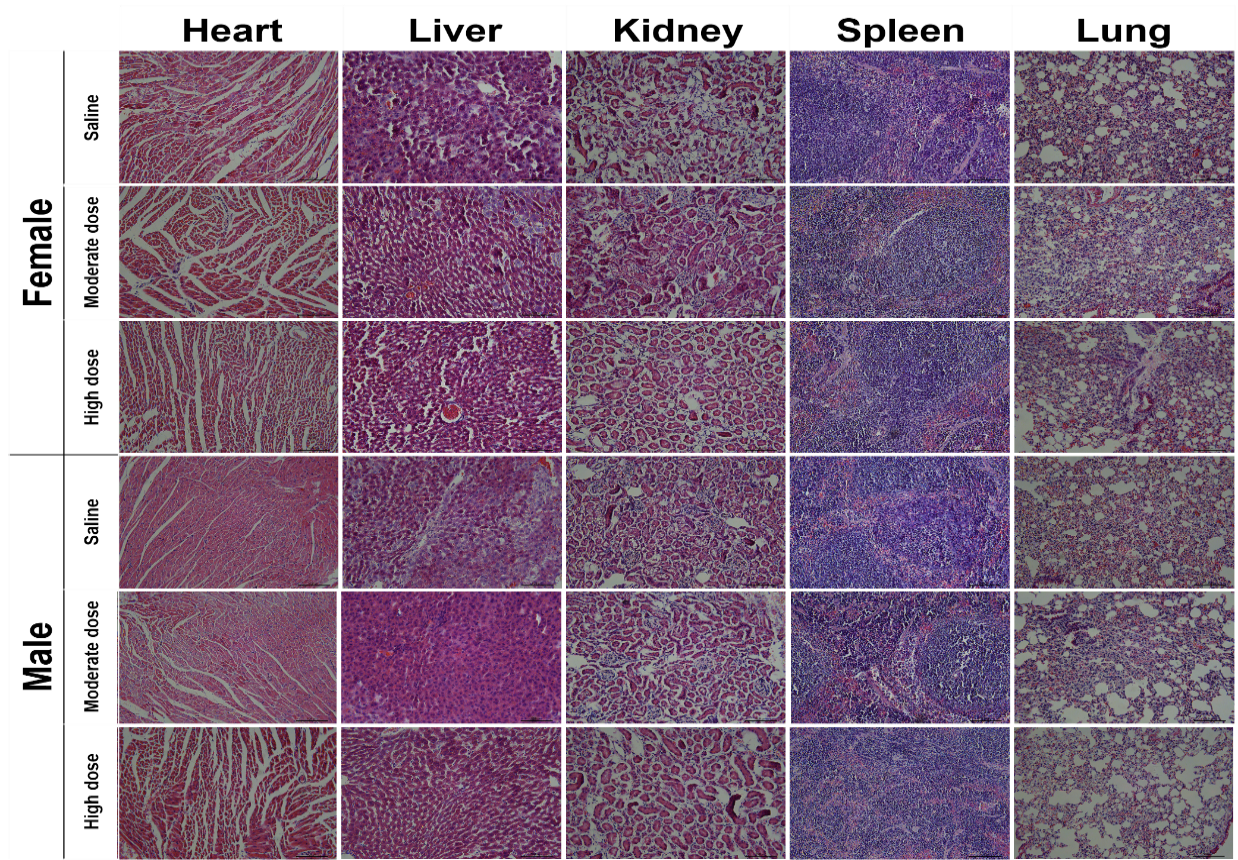
**

Figure. S4.

**HE staining of major tissues from mice injected with saline and different doses of ^99m^Tc-RWY.** Mice in the moderate dose group were injected with185 MBq/kg^99m^Tc-RWY, and mice in the high dose group were injected with 1850 MBq/kg^99m^Tc-RWY (normal dose: 74 MBq/kg calculated according to the species). Compared with the histology in the saline group, no histological changes were observed in the major organs of mice injected with the excess imaging dose.


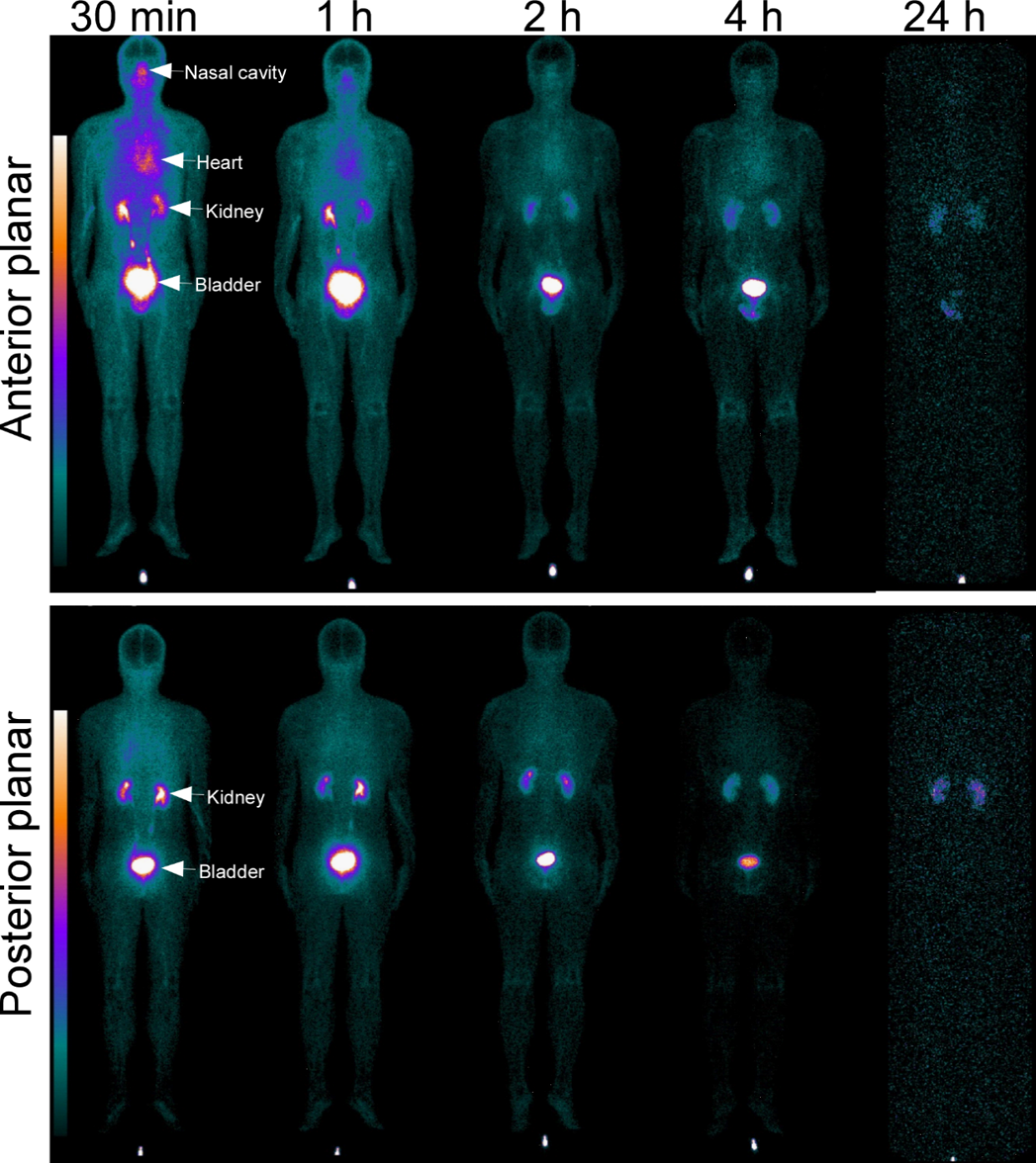


Figure. S5.

Anterior (upper row) and posterior (lower row) planar whole-body SPECT images of a representative subject showed the distribution of ^99m^Tc-RWY between 30 min and 24 hp.i. The predominant uptake of ^99m^Tc-RWY was observed in the bladder and kidney (white arrow). Apparent radiotracer uptake was also observed in the nasal cavity and heart at the early time point (30 min p.i.) and was almost undetectable at 2, 4, and 24 h after injection. The tracer accumulation in normal chestwas as low as background signals in all subjects.


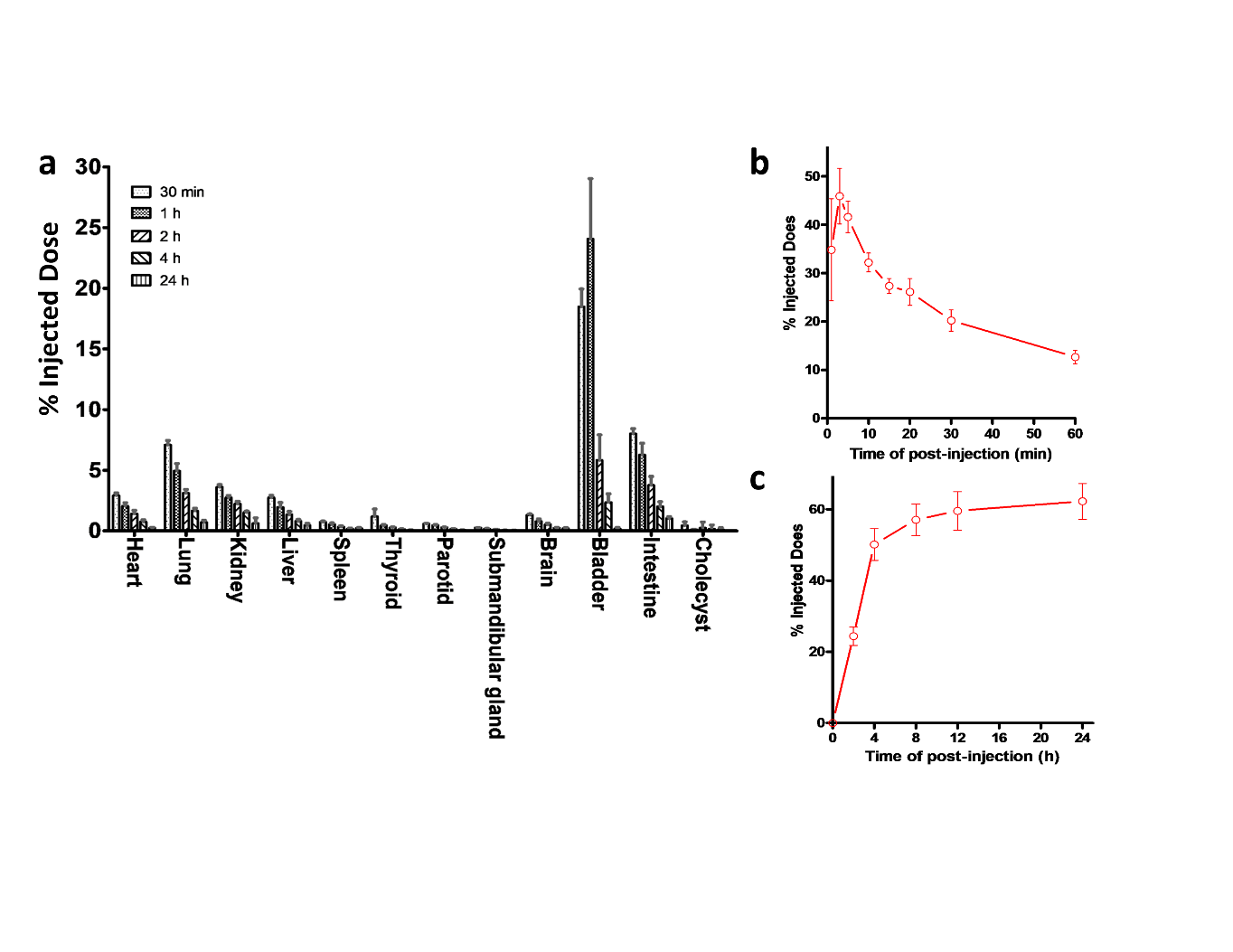


Figure. S6.

Quantitative analyses of ^99m^Tc-RWY in major organs of healthy volunteers (a). Region of interests (ROIs) drawn on anterior and posterior images were calculated from the whole-body images at 30 min, 1 h, 2 h, 4 h and 24 h p.i. The activities of ^99m^Tc-RWY in twelve visceral organs (heart, lung, kidney, liver, spleen, thyroid, parotid, submandibular gland, brain, bladder, intestine and gallbladder) were measured. The activities are expressed as the %ID. The averaged time-activity curves of ^99m^Tc-RWY in blood (b) and in urine (c) were measured in seven healthy volunteers. Error bars indicate standard deviations.


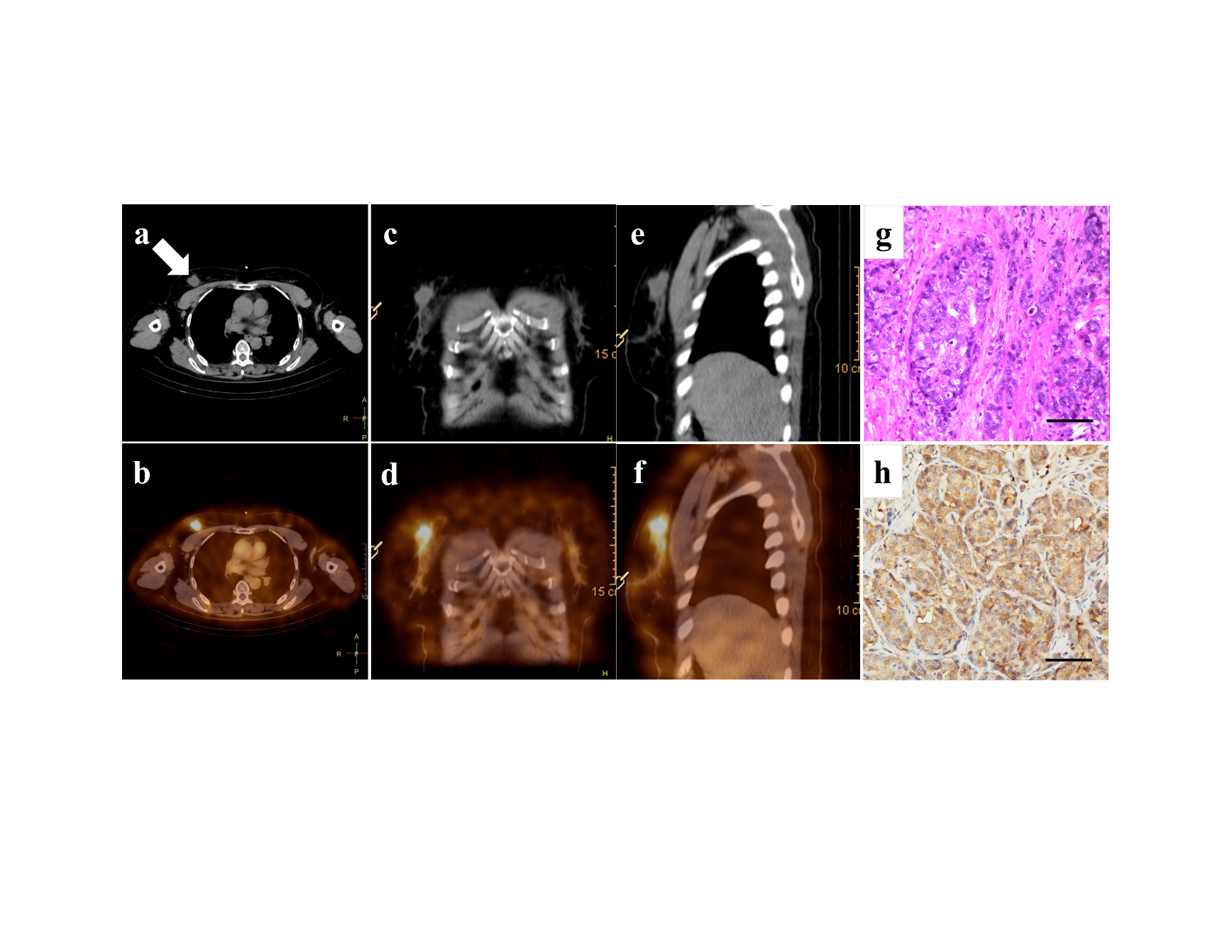


Figure. S7.

^99m^Tc-RWYSPECT/CT imaging of Patient #2, a 51-y-old woman with clinical stage III breast cancer in the left breast. (a**&d**) Transverse plane CT and SPECT/CT. (**b&e**) Coronal plane CT and SPECT/CT. (**c&f**) Sagittal plane CT and SPECT/CT. The tumor in the left breast showed much higher uptake compared with the normal breast tissues. The samples obtained from the high radioactivity area were developed with HE staining (**g**) and integrin α6 immunohistochemical staining (**h**). Both of them showed positive.

| **Volunteer No./Sex** | **Age (y)** | **Height(cm)** | **Weight (kg)** | **Body Mass Index (kg/m^2^)** | **Injected Activity (MBq)** |
| --- | --- | --- | --- | --- | --- |
| 1/M | 36 | 174 | 68 | 22.46 | 708.92 |
| 2/M | 35 | 174 | 72 | 23.78 | 838.42 |
| 3/M | 23 | 187 | 79.5 | 22.73 | 904.65 |
| 4/M | 26 | 178 | 87 | 27.46 | 966.44 |
| 5/F | 25 | 158 | 60 | 24.03 | 732.23 |
| 6/F | 29 | 160 | 54 | 21.09 | 686.72 |
| 7/F | 26 | 158 | 53 | 21.23 | 633.81 |
| Mean±SD | 28.57±5.06 | 169.86±11.35 | 67.64±12.87 | 23.26±2.17 | 781.60±123.22 |

Table S1.

**Demographic and clinical characteristics of healthy volunteers.** The seven healthy volunteers consisted of four males and three females aged between 23 and 36 years old.

| **Target Organ** | **Dosimetry data (×10^-3^ mSv/MBq)** | | |
| --- | --- | --- | --- |
|  | **Male (n = 4)** | **Female (n = 3)** | **Total (n = 7)** |
| Adrenal glands | 3.33±0.22 | 4.23±0.28 | 3.71±0.53 |
| Brain | 1.04±0.11 | 1.28±0.15 | 1.14±0.17 |
| Breasts | 1.67±0.13 | 2.08±0.15 | 1.84±0.25 |
| Gallbladder wall | 4.49±0.60 | 5.61±0.37 | 6.67±0.76 |
| Lower region of colon | 13.80±1.61 | 18.77±1.96 | 15.93±3.10 |
| Small intestine | 7.91±0.91 | 11.09±1.14 | 9.27±1.93 |
| Stomach wall | 9.82±1.17 | 13.87±1.62 | 11.55±2.50 |
| Upper colon | 10.94±1.37 | 15.30±1.73 | 12.81±2.72 |
| Heart wall | 7.76±1.49 | 9.16±1.23 | 8.36±1.47 |
| Kidneys | 9.81±0.36 | 11.63±0.59 | 10.59±1.06 |
| Liver | 3.10±0.21 | 3.65±0.30 | 3.34±0.37 |
| Lungs | 4.45±0.62 | 5.88±0.50 | 5.06±0.93 |
| Muscle | 2.43±0.18 | 3.07±0.18 | 2.71±0.38 |
| Ovaries | - | 7.74±0.52 | 7.74±0.52 |
| Pancreas | 4.29±0.41 | 5.61±0.47 | 4.86±0.81 |
| Red marrow | 2.58±0.19 | 3.22±0.21 | 2.85±0.39 |
| Osteogenic cells | 5.89±0.37 | 7.35±0.47 | 6.51±0.87 |
| Skin | 1.42±0.09 | 1.72±0.10 | 1.54±0.18 |
| Spleen | 4.45±0.15 | 6.16±0.82 | 5.18±1.03 |
| Testis | 2.38± 0.15 | - | 2.38±0.15 |
| Thymus | 2.56±0.25 | 3.09±0.23 | 2.79±0.36 |
| Thyroid gland | 12.08±1.62 | 14.83±3.19 | 13.26±2.62 |
| Urinary bladder wall | 19.15±2.58 | 31.70±3.11 | 20.38±6.92 |
| Uterus | - | 7.48±0.23 | 7.48±0.23 |
| Whole body | 2.74±0.20 | 3.33±0.32 | 2.99±0.39 |

Table S2.

**Dosimetry data of ^99m^Tc-RWY in healthy volunteers (n = 7).**

|  |  | Patient 1 | | | Patient 2 | | |
| --- | --- | --- | --- | --- | --- | --- | --- |
| Clinical lab | Normal range | Before injection | After injection | Change | Before injection | After injection | Change |
| WBC（10^9^/L） | 4.00-10.00 | 7 | 7.4 | 0.4 | 5.43 | 6.11 | 0.68 |
| HGB (g/L) | 110.0-150.0 | 77 | 71 | -6 | 125 | 128 | 3 |
| HCT (L/L) | 0.370-0.480 | 0.224 | 0.214 | -0.01 | 0.38 | 0.391 | 0.011 |
| PLT (10^9^/L) | 100-300 | 301 | 257 | -44 | 311 | 352 | 41 |
| RBC (10^12^/L) | 3.50-5.00 | 2.38 | 2.35 | -0.03 | 4.03 | 4.08 | 0.05 |
| Na^+^ (mM) | 136-145 | 141 | 140 | -1 | 139.2 | 140.8 | 1.6 |
| K^+^ (mM) | 3.50-5.20 | 3.77 | 3.54 | -0.23 | 4.3 | 4.3 | 0 |
| Cl^-^ (mM) | 96.00-108.00 | 107.8 | 107 | -0.8 | 104.4 | 103.7 | -0.7 |
| CO_2_(mM) | 22.00-31.00 | 19.7 | 20.5 | 0.8 | 24.8 | 28.85 | 4.05 |
| BUN (mM) | 2.80-7.60 | 6 | 4.7 | -1.3 | 3.1 | 2.92 | -0.18 |
| Cr (uM) | 48.00-100.00 | 67.6 | 83.8 | 16.2 | 50.2 | 54.68 | 4.48 |
| AST (IU/L) | 8.00-40.00 | 31 | 32 | 1 | 27.9 | 28.88 | 0.98 |
| ALT (IU/L) | 5.00-40.00 | 22 | 22 | 0 | 40.1 | 40.63 | 0.53 |
| ALP (IU/L) | 30.00-120.00 | 77 | 81 | 4 | 92 | 100 | 8 |
| TB (uM) | 5.00-21.00 | 5 | 5.3 | 0.3 | 9.8 | 9.17 | -0.63 |

Table S3.

**Baseline and post procedural laboratories.** Average values of clinical labsperformed before and after administration of ^99m^Tc-RWY in two human subjects. WBC, white blood cell count; HGB, hemoglobin; HCT, hematocrit; PLT, platelet count; RBC, red blood cell count; Na^+^, sodium ion; K^+^, potassium ion; Cl^-^, chloride ion; BUN, blood urea nitrogen; Cr, creatinine; AST, aspartate aminotransferase; ALT, alanineaminotransferase; Alk, alkaline phosphatase; TB, total bilirubin.

| **Variables** | **Cases**  **(n = 92)** | **Integrin α6 expression** | | **P Value** |
| --- | --- | --- | --- | --- |
|  |  | **Low (IRS <4.0)** | **High (IRS >4.0)** |  |
| **Age (years)** |  |  |  | 0.043* |
| ＜50 | 50 | 18 (36.0%) | 32 (64.0%) |  |
| ≥ 50 | 42 | 24 (57.1%) | 18 (42.9%) |  |
| **Gender** |  |  |  |  |
| Female | 92 | 42 (45.7%) | 50 (54.3%) |  |
| **Tumor Size** |  |  |  | 0.882 |
| ≤ 2.5 cm | 38 | 17 (44.7%) | 1. (55.3%) |  |
| >2.5 cm | 54 | 25 (46.3%) | 29 (53.7%) |  |
| **Menopause** |  |  |  | 0.863 |
| No | 32 | 15 (46.9%) | 17 (53.1%) |  |
| Yes | 60 | 27 (45.0%) | 33 (55.0%) |  |
| **LN Infiltration** |  |  |  | 0.599 |
| No | 26 | 13 (50.0%) | 13 (50.0%) |  |
| Yes | 66 | 29 (43.9%) | 37 (56.1%) |  |
| **Tumor Location** |  |  |  | 0.157 |
| Left | 49 | 19 (38.8%) | 30 (61.2%) |  |
| Right | 43 | 23 (53.5%) | 20 (46.5%) |  |
| **ER Status** |  |  |  | 0.398 |
| Negative | 32 | 17 (53.1%) | 15 (46.8%) |  |
| Positive | 60 | 25 (41.7%) | 35 (58.3%) |  |
| **PR Status** |  |  |  | 0.791 |
| Negative | 43 | 19 (44.2%) | 24 (55.8%) |  |
| Positive | 49 | 23 (46.9%) | 26 (53.1%) |  |
| **Her-2 Status** |  |  |  | 0.48 |
| Negative | 60 | 29 (48.3%) | 31 (51.7%) |  |
| Positive | 32 | 13 (40.6%) | 19 (59.4%) |  |
| **TNBC** |  |  |  | 0.783 |
| Negative | 83 | 37 (44.6%) | 46 (55.4%) |  |
| Positive | 9 | 5 (55.6%) | 4 (44.4%) |  |
| **Histological Grade** |  |  |  | 0.722 |
| Moderately Differentiated (G1/G2) | 64 | 30 (46.9%) | 34 (53.1%) |  |
| Poorly Differentiated (G3) | 28 | 12 (42.9%) | 16 (57.1%) |  |
| **Tumor Status (T)** |  |  |  | 0.191 |
| T1 | 10 | 5 (50.0%) | 5 (50.0%) |  |
| T2 | 51 | 25 (49.0%) | 26 (51.0%) |  |
| T3 | 12 | 2 (16.7%) | 10 (83.3%) |  |
| T4 | 19 | 10 (52.6%) | 9 (47.4%) |  |
| **TNM Staging** |  |  |  | 0.882 |
| I-II | 38 | 17 (44.7%) | 21 (55.3%) |  |
| III-IV | 54 | 25 (46.3%) | 29 (53.7%) |  |

Table S4.

**Correlation between integrin α6 expression and clinicopathological variables in patients with breast cancer (n = 92).**
